# Physico-chemical characteristics of evaporating respiratory fluid droplets

**DOI:** 10.1098/rsif.2017.0939

**Published:** 2018-02-28

**Authors:** Eric P. Vejerano, Linsey C. Marr

**Affiliations:** 1 Center for Environmental Nanoscience and Risk, Environmental Health Sciences, Arnold School of Public Health, University of South Carolina, Columbia, SC 29208, USA; 2 Civil and Environmental Engineering, Virginia Tech, Blacksburg, VA 24061, USA

**Keywords:** influenza, phase separation, aerosol transmission, relative humidity, mucus, crystallization

## Abstract

The detailed physico-chemical characteristics of respiratory droplets in ambient air, where they are subject to evaporation, are poorly understood. Changes in the concentration and phase of major components in a droplet—salt (NaCl), protein (mucin) and surfactant (dipalmitoylphosphatidylcholine)—may affect the viability of any pathogens contained within it and thus may affect the efficiency of transmission of infectious disease by droplets and aerosols. The objective of this study is to investigate the effect of relative humidity (RH) on the physico-chemical characteristics of evaporating droplets of model respiratory fluids. We labelled these components in model respiratory fluids and observed evaporating droplets suspended on a superhydrophobic surface using optical and fluorescence microscopy. When exposed to continuously decreasing RH, droplets of different model respiratory fluids assumed different morphologies. Loss of water induced phase separation as well as indication of a decrease in pH. The presence of surfactant inhibited the rapid rehydration of the non-volatile components. An enveloped virus, *ϕ*6, that has been proposed as a surrogate for influenza virus appeared to be homogeneously distributed throughout the dried droplet. We hypothesize that the increasing acidity and salinity in evaporating respiratory droplets may affect the structure of the virus, although at low enough RH, crystallization of the droplet components may eliminate their harmful effects.

## 1. Introduction

There is growing evidence that transmission of some infectious diseases via the airborne route is important [1–5]. Furthermore, there appears to be a relationship between transmission and environmental conditions for a subset of diseases. For influenza, multiple lines of evidence, including epidemiological studies [6–8] and laboratory studies with animal models [9,10] suggest that there is a relationship between incidence or transmission and humidity. Additionally, studies of influenza virus viability in droplets and aerosols have shown that the virus survives well at low relative humidity (RH), below approximately 50%; results are inconsistent at higher RH [11–14].

What remains unclear is how humidity could affect virus viability. A common misperception is that airborne viruses are naked particles floating around in air. However, when released from the host, whether through coughing, sneezing, talking, or normal breathing, the virions are immersed in respiratory fluid. If the virion itself is not in direct contact with air, then how could its viability be affected by humidity?

Some models of the transport and viability of airborne viruses have assumed that the carrier liquid can be modelled as water [15,16]. This is an oversimplification that ignores the complex composition of respiratory fluid. For example, a 60 µm droplet has a volume of approximately 0.1 nl and would contain approximately 1 virion if the virus concentration in the bulk liquid were 10^7^ ml^−1^, if the bulk concentration also applied to the droplet. Assuming a simplified model of respiratory fluid composition [17–22], the droplet would contain approximately 1 ng of salt, approximately 1 ng of total protein and approximately 0.06 ng of surfactant. The mass of each component is at least five orders of magnitude larger than the mass of the virion. These components form the virion's microenvironment and should not be disregarded.

When expelled from the respiratory system, droplets are subject to an abrupt change in RH from approximately 100% to ambient conditions. In all but the most humid environments, the droplets evaporate quickly to approximately half their initial diameter [23,24], and concomitantly, the concentrations of salts, proteins and other components increase by nearly an order of magnitude due to the loss of water. Evaporation can induce a variety of physico-chemical transformations in the droplet, as has been shown for atmospheric aerosol particles. For example, aerosol particles containing salt and organic material undergo liquid–liquid phase separation, forming a core-shell structure, when exposed to low RH [25]. Furthermore, the pH of aerosol particles varies with degree of phase separation and with RH [26,27]. These types of transformations in respiratory droplets could have important implications for the viability of any pathogens contained inside the droplets.

While the physical properties of respiratory droplets in ambient air have dominated discussion of airborne transmission, the chemical properties of the droplets have often been neglected. The chemical microenvironment immediately surrounding virions in droplets and aerosols is likely to be an important determinant of their stability. Evidence for this claim stems from our previous work showing that influenza virus viability is inversely correlated with salt concentration in evaporating droplets of media containing negligible amounts protein [11]. Virus viability is known to depend on salt concentration and pH [14,28–30], among other factors.

The extent of evaporation of respiratory droplets, and thus the concentrations of various components of the fluid, is determined by RH. Thus, determining the effect of RH on droplet composition is essential in understanding viral infectivity and transmission via droplets and aerosols. The objective of this study is to investigate the effect of RH on the physico-chemical characteristics of droplets consisting of model respiratory fluids and the enveloped virus *ϕ*6.

## 2. Material and methods

### 2.1. Generation and control of humid air

To control the RH in the chamber, we mixed dry clean air and humid air at various mixing ratios that were precisely controlled by mass flow controllers. We generated humid air by bubbling dry, clean air through a 1 l bottle filled with approximately 0.90 l of (18 *m*Ohm) water at approximately 22°C. We then directed the pre-saturated air stream through two 76 l containers that were connected in series, each containing 3.8 l of water. We then directed the air stream through a 1 l container heated at approximately 35°C and finally through a 1 l empty container at approximately 22°C to condense excess moisture. We measured RH using a micro-humidity sensor (Dracal Technologies) equipped with a sensor that compensated for temperature (SHT75, Sensirion Inc.). We periodically calibrated the sensor using a water-saturated NaCl solution (76% RH) at approximately 22°C as a one-point calibration standard. We fixed the total flow rate of the mixed air stream to 1 l min^−1^. We directed only 25 ml min^−1^ of the humidified air into the chamber while we vented the remainder.

### 2.2. Chamber

We customized an FC310 chamber (Biosurface Technologies Inc.) to accommodate an 18 mm glass coverslip. We sealed the chamber using a 25 mm glass coverslip with a silicon gasket. We secured the 18 mm coverslip onto the recessed groove on the chamber using a small amount of high-vacuum grease. The average distance from the surface of the 18 mm superhydrophobic glass coverslip to the surface of the 25 mm glass coverslip that sealed the chamber was approximately 1200 µm as measured by a confocal microscope. We set the flow rate inside the chamber to 25 ml min^−1^, which translates to a linear velocity of 0.0064 m s^−1^.

### 2.3. Composition of simulated respiratory/saliva solution

We generated droplets from an aqueous solution containing NaCl (9 g l^−1^, physiological concentration), mucin (3 g l^−1^ porcine gastric mucin type III, Sigma-Aldrich^®^), and 1,2-dihexadecanoyl-*sn*-glycero-3-phosphocholine (DPPC, 0.5 g l^−1^, Avanti Inc.). DPPC is among the most abundant phospholipid lung surfactants that reduces surface tension during breathing [31]. We investigated phase transition and evaporation for droplets with (4C) and without (3C) surfactant. One of the authors donated the saliva samples. In this study, we measured gross changes only and not specific reactive sites on the mucin; we, therefore, used porcine gastric mucin type III as a surrogate for human mucin, which has similar mechanical properties [32].

### 2.4. Preparation of superhydrophobic substrates

We modified Teflon filters (pore size 0.2 µm) and 18 mm round glass coverslips to make their surfaces superhydrophobic. First, we dispersed 200 mg of silica nanoparticles (a gift from Evonik^®^, average particle size 14 nm) in 50 ml acetone. We dispersed the resulting suspension to minimize the size of the aggregated nanoparticles by sonicating for 5 min. We used 100 µl of the suspension and drop-casted onto polyvinylidene fluoride filters or glass coverslips. We then dried the filters at room temperature for at least 3 h before use while we dried the glass coverslips at 100°C for 5 h. We measured the contact angle of the droplet on the superhydrophobic surface. We measured the contact angle using a confocal microscope (Witec Corp.).

### 2.5. Formation of droplets on a superhydrophobic surface

We deposited droplets by holding filters or coverslips in front of the outlet of a TSI 3076 atomizer in a near-saturated chamber. The atomizer contained 5 ml of the mucin solution or real saliva and ran for 25 s. Aerosolization and impaction created submicron particles that eventually coalesced into supermicron droplets on the surface.

### 2.6. Labelling of mucin and staining of DPPC

We visualized mucin by labelling it with an Atto590 *N*-hydrosuccinimide ester dye (*λ*_ex_\*λ*_em_ = 594/624 nm, Sigma-Aldrich^®^). Briefly, we added 100 mg of mucin in 20 ml phosphate buffer (100 mM pH 8.5) to 1 mg of the dye dissolved in amine-free dimethylformamide (Molecular Biology Grade, Sigma-Aldrich^®^). We continually stirred the solution for 2 h at 22°C. Then, we added nine parts by volume of previously cooled aqueous ethanol solution (90% *v/v*) at −80°C to the suspension to induce precipitation of the mucin. We precipitated mucin overnight by maintaining the temperature of the suspension at −20°C. We recovered the precipitate by centrifuging the suspension at 50 000*g* for 15 min. We then washed the precipitate with cold ethanol (−80°C) to remove as much of the residual Atto590 dye, which is highly soluble in ethanol, as possible. We resuspended the dried mucin in 20 ml nanopure water and dialysed it overnight to remove phosphate ions that originated from the buffer. To visualize DPPC, we added 100 µg boron-dipyrromethene-phosphocholine (BODIPY-PC, *λ*_ex_\*λ*_em_ = 488\503 nm, Molecular Probes, Life Technologies Inc.) or 1-palmitoyl-2-{6-[(7-nitro-2-1,3-benzoxadiazol-4-yl)amino]hexanoyl}-*sn*-glycero-3-phosphocholine (NBD-PC, *λ*_ex_\*λ*_em_ = 460\534 nm, Avanti Polar Lipids Inc.) to unlabelled DPPC.

### 2.7. Tracking *ϕ*6 virus

We used *ϕ*6 bacteriophage propagated in the host *Pseudomonas syringae* as a surrogate for influenza virus. *ϕ*6 is enveloped and, at approximately 75 nm in diameter [33], is similar in size. To visualize the virus, we used 1-palmitoyl-2-{6-[(7-nitro-2-1,3-benzoxadiazol-4-yl)amino]hexanoyl}-*sn*-glycero-3-phosphocholine (NBD-PC, Avanti Polar Lipids Inc.), which is assumed to associate with the virus' lipid membrane because of its similar composition.

### 2.8. Microscopy

To visualize the droplets and the associated components, we used confocal (Witec Corp.) and fluorescence and bright-field (Axioskop 2 plus, Carl Zeiss Inc.) optical microscopes that were equipped with a 50× Carl Zeiss objective (LD = 9 mm, NA = 0.55). We used a rhodamine filter to observe the fluorescence of the mucin that was tagged with Atto590 dye while we used a FITC filter for BODIPY/NBD-PC. We captured all images at the green and red channels using a similar exposure time and composited images.

### 2.9. Evaporation of water

We measured the evaporation rate of water from droplets of different composition. The other droplet components are not volatile under ambient conditions. We used a bright-field optical microscope to measure the change in diameter of highly spherical droplets (at least appearing circular when viewed in the XY plane) at different RHs. For each RH, we observed 10–30 droplets and tracked their size. We used the AxioVision software (Carl Zeiss, Inc.) to determine the diameter of the droplet. The dimensionless droplet diameter is defined as the diameter at any given time divided by the initial diameter (*D*/*D*_o_). We quadratically fit *D/D*_o_ *v*. *time* and determined the slope at each *D/D*_o_ to extract the instantaneous evaporation rate, assuming that the density of the droplet remained constant. In most cases, the values at *D/D*_o_ < 0.4 were extrapolated from the best-fit lines, since we ceased measurement when *D/D*_o_ ∼ 0.4.

## 3. Results

We investigated the effect of RH, mediated through evaporation of water, on the physico-chemical characteristics of droplets of model respiratory solutions, whose composition is listed in table 1. We used NaCl, mucin, and DPPC to represent salts, proteins, and surfactants, respectively [18,34,35]. We isolated the effect of surfactant by considering droplets containing NaCl, mucin, and water (three components, 3C) and droplets containing the same three components plus DPPC (four components, 4C). The 3C and 4C bulk solutions were opaque and had a pH of 3.7, whereas real human saliva was clear and had a pH of 7.5.

**Table 1.** Concentration (g l^−1^) of the components used in model respiratory fluids. nm, not measured.

sample	NaCl	mucin	DPPC
three-component (3C)	9	3	0
four-component (4C)	9	3	0.5
human saliva (HS)	nm	nm	nm

### 3.1. Droplet size and morphology

The solutions were aerosolized, and droplets were deposited on superhydrophobic substrates under saturated conditions. The initial diameter of droplets ranged from 10 µm to 40 µm, with a mean of approximately 22 µm (electronic supplementary material, figure S1). The contact angle of an approximately 40 µm droplet was 154° ± 1° (electronic supplementary material, figure S2). The mucin and DPPC were labelled with red and green fluorescent dyes, respectively, to facilitate their localization in the droplets. Droplets exhibited different morphologies and composition from one to the next, as shown in the electronic supplementary material, figure S3, likely to be due to the low solubility of the components. Some droplets contained a relatively large amount of mucin and a relatively small quantity of surfactant, while others consisted largely of surfactant. A majority of the 4C droplets, however, resembled the one in the centre of electronic supplementary material, figure S3, containing both mucin and surfactant.

### 3.2. Droplet evaporation rates

**Figure 1.**
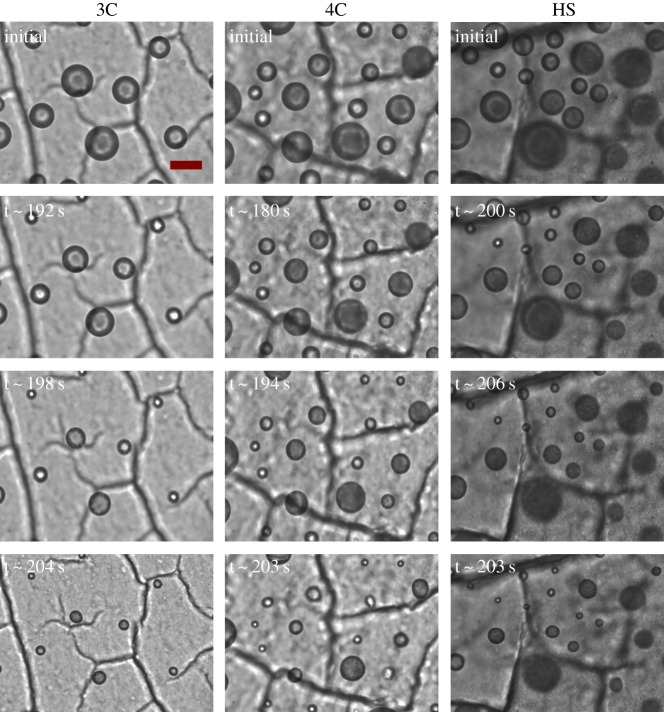
Representative bright-field optical images of evaporating droplets containing different components exposed at constant RH of 60%. Scale bar represents 20 µm. (Online version in colour.)

We transferred the droplets from saturated conditions to seven different values of RH ranging from 29% to 95% and measured the evaporation rate while RH remained constant. Because droplets were in contact with a superhydrophobic surface and did not experience air flow, results can only be interpreted qualitatively in applying them to airborne droplets under real environmental conditions. Figure 1 display representative images of evaporating droplets containing different components exposed at a constant RH of 60%. The droplets ranged in size from 10 µm to 40 µm, and changes in size were subtle for the first approximately 3 min (180 s). Over the next approximately 30 s, the droplets shrank noticeably to less than half their initial diameter for 3C droplets and to greater than half their initial diameter for 4C and HS droplets.

**Figure 2.**
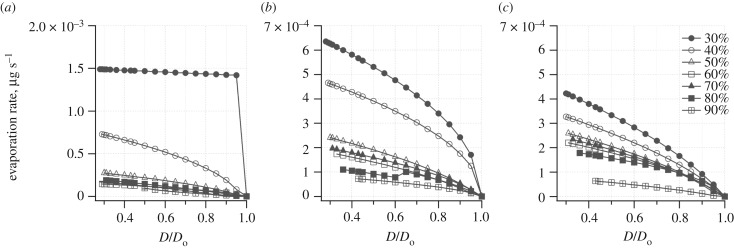
Evaporation rate of droplets at different RHs as a function of dimensionless droplet diameter (*D*/*D*_o_). (*a*), (*b*) and (*c*) are plots obtained from droplets aerosolized from 3C, 4C and HS solutions, respectively. At each RH, the minimum *D/D*_o_ value corresponds to the equilibrium diameter (*D*_eq_) calculated according to the Kohler equation or to the diameter of the solids in the case of RH lower than the efflorescence RH (ERH) of NaCl (approx. 44%). The legend for each RH is indicated in *c*. RH was held constant at the value indicated for that curve. Evaporation rates were obtained for droplets in contact with a superhydrophobic surface and without ventilation. Hence, results can only be interpreted qualitatively in applying them to airborne droplets under real environmental conditions.

Figure 2*a*–*c* depicts the evaporation rate of droplets of different composition at different RHs as a function of *D*/*D*_0_. We removed error bars in the trace for the sake of legibility. The relative standard deviation (RSD) ranged from approximately 2% to 25%; as the droplets shrank and became less spherical, the RSD became larger. As expected, the evaporation rate was higher at lower RH because of the larger driving force established by the difference between the actual RH and the equilibrium RH at the droplet surface. The presence of surfactant substantially affected the evaporation rate. It was two to ten times faster for 3C droplets compared to 4C and human saliva (HS) droplets that contained surfactant. For 3C droplets, the evaporation rate was much faster at 30% and 40% RH than at higher RHs. For 4C droplets, the difference at 30% and 40% RH was less pronounced, and for HS droplets, the evaporation rates were even more similar as a function of RH. The evaporation rate increased as droplet size shrank.

Real airborne respiratory droplets are expected to shrink to their equilibrium size in less than a second [24], substantially faster than observed for the droplets in this study, in which mass transfer was limited by the relatively more quiescent conditions and the presence of the substrate that would allow for locally elevated RH near the droplet/surface interface. The average evaporation rate of a suspended 22 µm droplet that shrinks to half its size in 0.8 s is approximately 7 × 10^−3^ µg s^−1^, which is almost 10 times higher compared to our droplets that rested on a superhydrophobic surface. Droplets containing ammonium sulfate and similar in size to ours reached their equilibrium size in approximately 4 s when exposed to 50% RH and approximately 20 s when exposed to 85% RH [36].

### 3.3. Effect of decreasing relative humidity on morphology of 3C and 4C droplets

The transformations of 3C and 4C droplets exposed initially to 95% RH and then subjected to a 5%/min decrease in RH differed. These results pertain to droplets as they were drying on a surface under conditions of decreasing RH. The RH ramp may or may not have been sufficiently slow for results to be representative of airborne droplets that have reached equilibrium, but due to the influence of the surface, these droplets would continue to evaporate to dryness (i.e. their equilibrium in this experimental system) at any RH.

**Figure 3.**
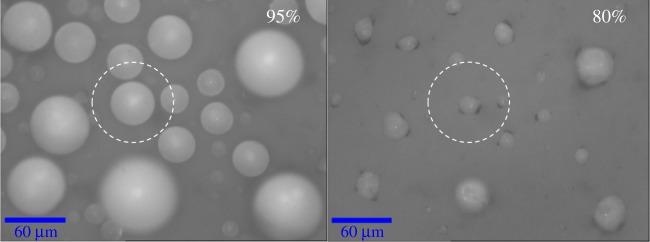
Optical confocal image of 3C droplets exposed initially at 95% RH, ramped down to 80% at 1%/min. The droplets became transparent at 80% RH. (Online version in colour.)

Figure 3 shows that 3C droplets became transparent when the RH was ramped down to 80% at a rate of 1%/min. For this experiment, we used a ramping rate of 1%/min to increase the resolution of RH at which an observable conformational change (i.e. droplet opacity) in mucin occurred. Electronic supplementary material, figure S5 depicts the complete series of images. Images in figure 3 and the electronic supplementary material, figure S5 are two-dimensional projections that were optically sectioned at the maximum droplet diameter by a confocal microscope.

**Figure 4.**
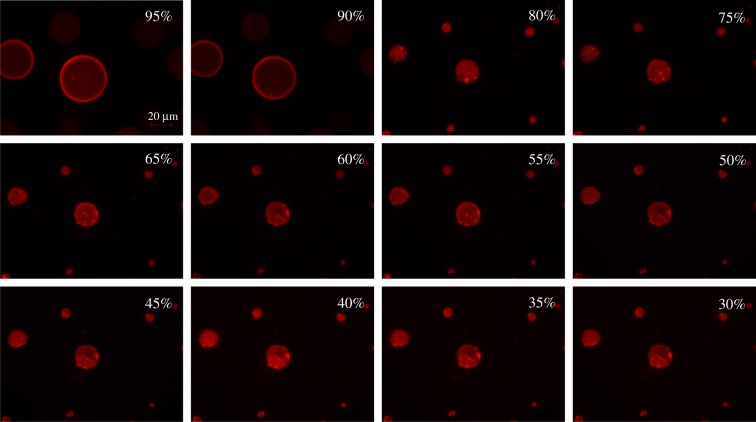
Fluorescence images of droplets exposed to decreasing RH. Droplets were aerosolized from a solution containing NaCl and mucin in water. The red colour indicates mucin. Droplets were initially exposed at 100% RH, and then the RH was ramped down at 5%/min. Droplets exposed at near-100% RH appeared similar to those at 95%. Scale bar represents 20 µm.

**Figure 5.**
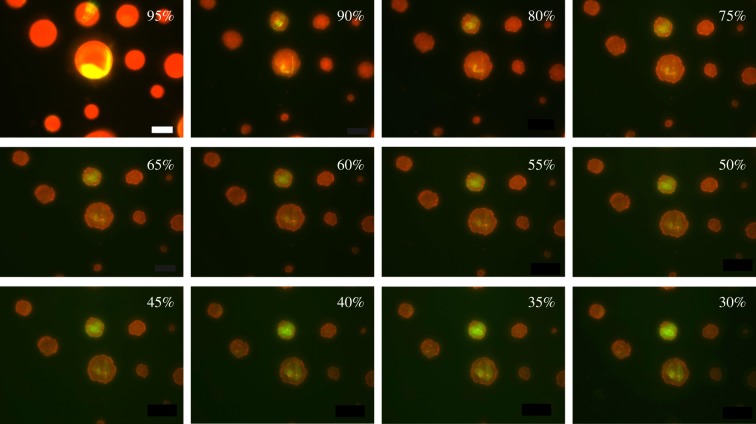
Fluorescence images of droplets exposed to decreasing RH. Droplets were aerosolized from a solution containing NaCl, mucin and DPPC in water. The red and green colours indicate mucin and DPPC, respectively. Droplets were initially exposed at 100% RH, and then the RH was ramped down at 5%/min. Droplets exposed at near-100% RH appeared similar to those at 95%. Scale bar represents 20 µm.

Figures 4 and 5 show time series of evaporating 3C and 4C droplets, respectively, at certain RHs; these images are representative of three experimental runs for each type of droplet. The 3C droplets exhibited phase separation with a core-shell structure. At 95% RH, the shell consisted of a thin layer of mucin indicated by the red ring around the droplet with a few protein aggregates visible as highly intense red particles. The remaining mucin was localized in the core, which consisted of smaller and fewer mucin aggregates. As RH decreased, the droplets shrank dramatically between 95% and 80% RH, and the shell disappeared while the number of protein aggregates increased indicating phase transition [37,38]. As the droplets continued to evaporate at RH < 80%, salt started to crystallize at 60% RH and finally appeared as a near-cubic structure exhibiting a partially engulfed morphology at 50% RH and lower. The solid structure visible at 60% RH was an NaCl crystal; if this structure had been mucin aggregates, we would have observed an intense red fluorescence instead. The onset of salt crystallizing above the deliquescence RH may be due to mucin inducing contact efflorescence [39]. The intense red fluorescence observed for the dried droplet resulted from the increasing amount of transmitted excitation light reaching the labelled mucin.

In contrast to 3C droplets, 4C droplets initially exhibited a homogeneous structure with a single aqueous phase (i.e. no core-shell structure) (figure 5). While all droplets had a few small aggregates of suspended mucin, some also had large cylindrical crystals of DPPC. Although NaCl can crystallize into different shapes [40], it is not expected to form cylindrical crystals under the conditions used here. The DPPC crystals appeared as a fuzzy yellow structure in figure 5 at 95% RH; the fuzziness was due to the crystals moving faster than the camera's exposure time. As RH decreased at a rate of 5%/min, DPPC and salt started to crystallize at the centre of the droplets until they finally formed a fully engulfed morphology. In these droplets, most of the shrinkage took place between 95% and 90% RH.

In addition to visualizing droplets changing dynamically while RH decreased continuously, we also investigated step changes in RH from approximately 95% to 50% and from approximately 95% to 29%. We acquired images 30 s after the step change in RH (electronic supplementary material, figure S4). Even though the droplets did not have sufficient time to shrink as much as during the ramp experiments, the morphologies were similar in both cases (step change in RH versus ramped RH). The droplets were not at equilibrium, as they would have continued to evaporate to dryness, as discussed below.

### 3.4. Re-absorption of water

To determine the effect of solution composition on the uptake of water, we exposed desiccated droplets that had equilibrated at 29% RH to a step change to approximately 100% RH. The 3C droplets readily absorbed water after approximately 30 s and became nearly spherical again but were smaller than the original droplets. However, the 4C droplets containing surfactant did not absorb water, even after 10 min of exposure at approximately 100% RH.

### 3.5. Virus localization

**Figure 6.**
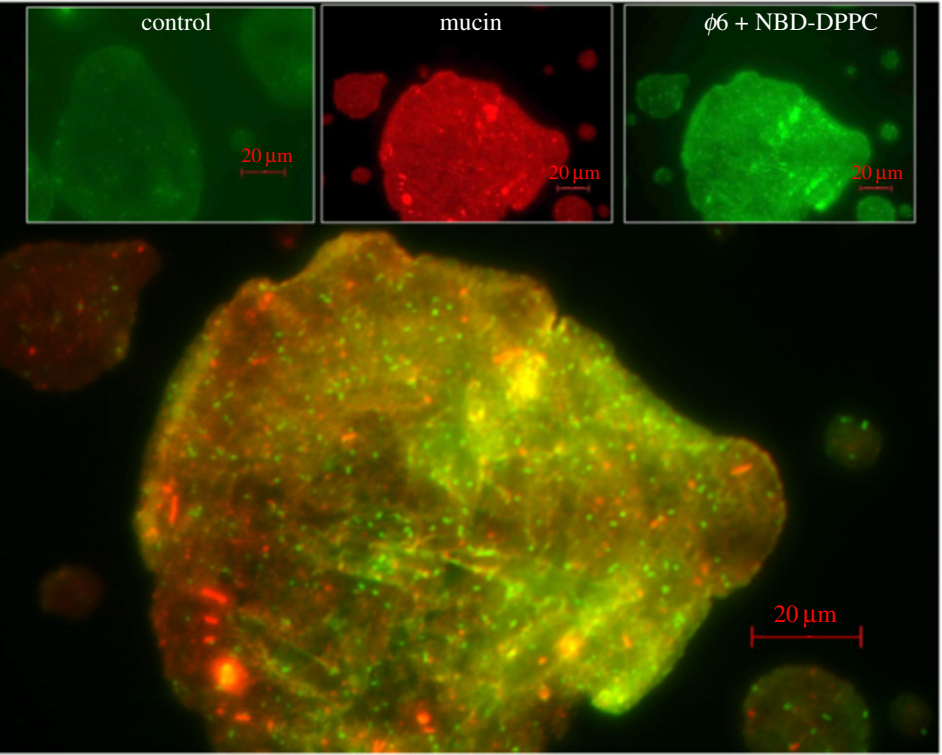
Composite fluorescent image of a 4C droplet containing *ϕ*6 virus exposed to 29% RH. The bright green dots approximately 1 µm in size may indicate the location of the virus. All scale bars are 20 µm.

Pinpointing the location of a viral pathogen in the droplets is important to understand its chemical microenvironment and thus its susceptibility to inactivation. The addition of fluorescently tagged lipid, NBD-PC, assumed to partition to the lipid membrane of the enveloped virus *ϕ*6 that was spiked into the solution, produced highly fluorescent approximately 1 µm dots that were dispersed throughout the droplet shown in figure 6. The initial size of the droplet was approximately 200 µm, and the number of fluorescent dots in the droplet was consistent with the estimated number of viral particles (approx. 200 particles) spiked into it. These fluorescent dots were not associated with any of the large mucin aggregates. In the control that did not contain any virus, only a few, weakly fluorescent dots were visible. Efforts are currently underway to label the virus with a suitable fluorophore to localize it in droplets with greater confidence.

## 4. Discussion

Sneezing and coughing expel a turbulent cloud of aerosols [41] whose size distribution spans more than two orders of magnitude [24,42,43]. Talking and breathing can also produce aerosols [44–46]. These aerosols are initially expelled from the respiratory tract at approximately 31°C [47] and nearly saturated RH (approx. 100%) [48]. Some of these are small enough to remain airborne for several minutes to hours, during which they equilibrate with ambient air, mainly by losing water if ambient RH is less than 100%. Our results show that as water was lost, a droplet underwent a variety of physico-chemical changes that could affect the stability of any pathogens contained in it.

### 4.1. Droplet transformation

A 3C droplet containing water, salt and mucin exhibited an initial core-shell morphology, in which the mucin separated from the other components and became concentrated at the air–liquid interface. For a droplet containing NaCl and water, once a salt nucleus forms and exceeds the critical size, the evaporation rate is expected to increase sharply [49]. However, we did not observe such an effect for the 3C and 4C droplets and attribute this behaviour at least partly to the presence of mucin and DPPC that were undergoing phase changes. For a 4C droplet that also contained the surfactant DPPC, its amphiphilic character allowed it to bind with salt and mucin and drive water from the core to the outer shell. As water evaporated, NaCl was enriched near the surface shell and then diffused toward the core. Several simulation studies performed on the interaction of a DPPC bilayer with NaCl indicate that Na^+^ binds strongly to the carbonyl group by replacing the water that is initially coordinated into this group, while Cl^−^ associates with the choline group of DPPC [29]. Ultimately, the dried droplet formed a nucleus of NaCl crystals associated with DPPC.

For comparison, You *et al*. report that droplets consisting of ammonium sulfate and oxygenated organic material, exposed to decreasing RH, undergo liquid–liquid phase separation with salt crystallizing in the centre [25]. Their droplets resemble a hybrid of our 3C droplets with a mucin shell at RH ≥ 90% and our 4C droplets with a crystallized core at RH ≲ 50%. Differences may be due to the presence of surfactant in our droplets. Characterizing the various components in a micron-scale droplet presents an experimental challenge. Ideally, the droplet would be suspended in air, as with optical tweezers, but such an approach is not compatible with the microscopy-based methods required for localizing different components. Therefore, we studied droplets on a surface. These droplets evaporated in minutes rather than the less than 1 s expected for respiratory aerosols to reach their equilibrium size [23,24]. The proximity of the droplets to one another on the surface might have inhibited air flow between them, creating regions with higher humidity between the droplets. Flow through the chamber was laminar, limiting mass transfer of water from the droplets to the bulk air, in contrast to the turbulent flow experienced by aerosolized respiratory droplets [41]. These conditions resulted in slower equilibration and a longer time for components in the droplets to rearrange and phase separate [50]. It is possible that rapid equilibration in aerosols could inhibit crystallization, leading to amorphous morphology instead. Additionally, all droplets at RH ≤ 90% eventually appeared to desiccate completely, even though the Kohler equation [51] predicts that they would retain some water. As the droplets evaporated, deposition of components on the substrate surface and capillary action may have promoted departure from sphericity and further loss of water.

Other limitations of this work are related to the composition of the solutions. To label respiratory fluid components for visualization, we necessarily synthesized a model respiratory fluid. Real respiratory fluid is far more complex, containing a variety of salts, proteins, surfactants and other components. The total protein content of respiratory fluid has been found to vary widely, ranging from 0.03 g l^−1^ in nasal fluid [52] to 85 g l^−1^ in alveolar fluid [53] of healthy individuals. The concentrations of these components surely vary between individuals and within individuals over time, especially if they have a respiratory infection. An infected individual's mucin levels may be elevated by a factor of 4 [54], potentially leading to significant differences in phase separation and other physico-chemical changes in his or her respiratory droplets.

There is indirect evidence of a change in pH in the droplets as RH decreases. The conformation of mucin depends on pH [55], and the change in transparency of 3C droplets at RH < 80% (figure 3) may signal the onset of gelation. The measured pH of the bulk 3C and 4C solutions was approximately 3.7. At pH > 2, mucin exists as a random coil [56]. However, at pH 2, carboxylate salt bridges on the mucin break, unfolding it and exposing the hydrophobic regions, which then cross-link to form a gel [56–58]. The onset of gelation suggests that the pH may have decreased below 2, at least in regions containing mucin. The rapid reabsorption of water by the 3C dessicated droplets further supports that mucin transformed into a gel or assumed a gel-like behaviour [59,60] compared to components that exhibit glassy behaviour [61,62]. Increasing ionic strength, as occurs in evaporating droplets, has been shown to produce conformational changes and aggregation but not gelation in mucin [63–65]. However, increasing ionic strength may aid gelation by initially breaking electrostatic attractions within the mucin. This effect may be tempered in real respiratory fluid droplets because of its higher pH and the presence of buffers.

Establishment of an equilibrium state (i.e. formation of insoluble crystals) inhibited the rapid absorption of water into the dried 4C droplet. A kinetic effect was unlikely [66] because at sufficiently high RH, water condensation on a glassy amorphous particle is expected to be rapid [67].

Our results indicate that a film of surfactant encapsulates the dried 4C droplets. Otherwise, exposure to saturated RH would have resulted in rapid swelling of regions with exposed mucin. This observation suggests that some fraction of the DPPC diffuses toward the surface or associates with the glycoproteins in mucin [68], while the other fraction associates with NaCl and forms the core of the dried droplet. The glycan structures in mucin [69] as well as the protein side chain that absorbs water may be covered by a film of surfactant rendering them unable to bind with water. We infer that in a dried droplet containing surfactant, carbonyl groups on the DPPC, which binds water, might already be occupied by tightly-bound Na^+^ ions [29].

### 4.2. Implications for virus stability

The widely reported relationship between viability of viruses in droplets and RH may be due to changes in physico-chemical characteristics as a droplet equilibrates with surrounding air. As discussed previously, there is interplay between ionic strength and pH, both of which are known to affect virus stability. If pH is indeed lower in the droplets, this could be a mechanism for inactivation of a pathogen, as we have proposed for influenza virus [30].

Determining the mechanism of inactivation of a virus in respiratory droplets requires understanding not only the physico-chemical microenvironment but also the precise location of the virion, as we have shown that the droplets are internally heterogeneous. In a 4C droplet, the virus appeared dispersed throughout the droplet or possibly on the surface. Some have proposed that processes at the air-liquid interface drive inactivation of viruses in droplets [4,70,71].

Enveloped viruses with a lipid membrane may be protected primarily by two components in respiratory fluid: mucin and DPPC. Previous studies have shown that at low RH, mucin protects the virus from damage by desiccation [72,73]. Changes in pH and ionic strength may expose hydrophobic domains of the mucin, allowing it to cross-link to form a gel [57]. Because of the hydrophobic nature of the viral envelope, the virus may associate with these exposed hydrophobic domains. However, we did not observe *ϕ*6 virus to be associated with mucin aggregates (figure 5).

If small droplets or aerosols are inhaled, rehydration should also be considered. They are subject to re-equilibration within the saturated environment of the respiratory system. Previous work has shown that abrupt rehydration inactivates non-enveloped virus [28] but not enveloped virus. However, our results with model respiratory fluids suggest that aerosols entering the nearly saturated humidity of the respiratory tract [48] will not undergo rapid rehydration because surfactant appears to inhibit reabsorption of water.

Aside from mucin, DPPC may protect the virus' lipid membrane. As the droplet dries, surfactant concentration increases. Because the viral membrane has similar composition, surfactant will likely partition to the virus' lipid membrane, acting as a ‘sacrificial’ viral coating and possibly protecting the virus' lipid membrane from structural damage. Interaction of DPPC with the surface proteins may increase the likelihood of being associated with the hydrophobic domains on the mucin gel, explaining the high viability of influenza A virus in human mucus [74]. Simulation of the interaction of DPPC with the BM2 channel of influenza B suggests that DPPC forms a lipid bilayer on the BM2 protein [75].

## 5. Conclusion

We assessed the detailed physico-chemical characteristics of respiratory droplets in ambient air exposed to different RH. Evaporation of water led to changes in the concentration and phase of major components in a droplet: salt, protein and surfactant. Droplets of different model respiratory fluids exposed to decreasing RH formed different morphologies. Loss of water induced the components to phase separate, and pH appeared to decrease. The non-volatile components of dried droplets that contained DPPC did not rehydrate rapidly when exposed to saturated RH. In a desiccated droplet, an enveloped virus, *ϕ*6, appeared to be homogeneously distributed. These results suggest that physico-chemical changes occurring in a droplet may affect the viability of any pathogens contained within it and thus may affect the efficiency of transmission of infectious disease by droplets and aerosols.

## Supplementary Material

Supplementary Materials

## Data accessibility

Electronic supplementary material, additional figures: Figure S1. Initial droplet size distribution obtained from aerosolizing the three solutions; Figure S2. Contact angle of a ∼40 µm droplet; Figure S3. Representative morphologies of droplets exposed to a step change in RH from 100% to 29%; Figure S4. Phase separation in 3C and 4C droplets exposed at very high RH (≥95%) followed by a step change to 50% or 29% RH; Figure S5. Sequence of confocal bright-field images for droplets exposed at different RH ramped down by 1% RH/min.
