# Supplementary material for: Physico-chemical characteristics of evaporating respiratory fluid droplets
Source: J R Soc Interface. 2018 Feb 28;15(139):20170939. doi: 10.1098/rsif.2017.0939 (PMC5832737; doi:10.1098/rsif.2017.0939)
Supplement: Supplementary Materials [file rsif20170939supp1.docx]

*Supplementary Materials*

*Journal of the Royal Society Interface*

Physico-Chemical Characteristics of Evaporating Respiratory Fluid Droplets

Eric P. Vejerano^a^ and Linsey C. Marr^b^

^a^Center for Environmental Nanoscience and Risk, Environmental Health Sciences, Arnold School of Public Health, University of South Carolina, Columbia, SC 29208; ^b^Civil and Environmental Engineering, Virginia Tech, Blacksburg, VA 24061

| 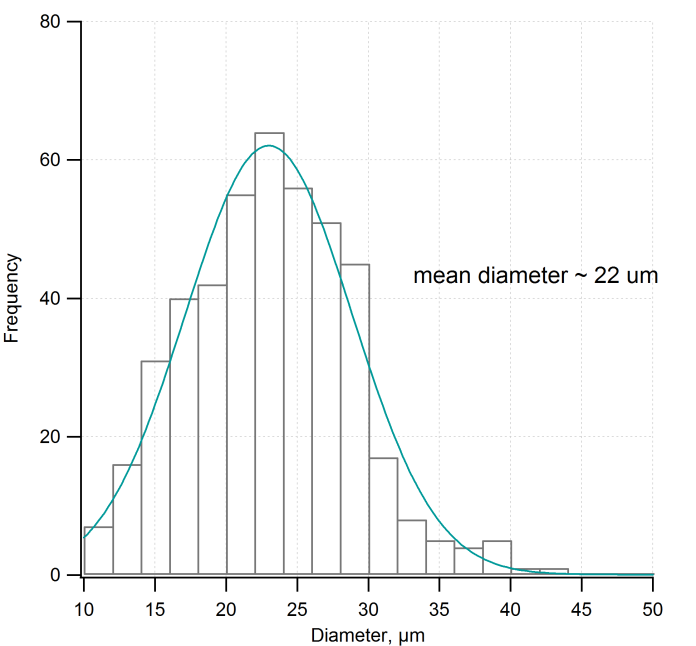  **Figure S1.** Initial droplet size distribution obtained from aerosolizing the three solutions. Only the droplets that were used for measuring the evaporation rate were counted. |
| --- |

| 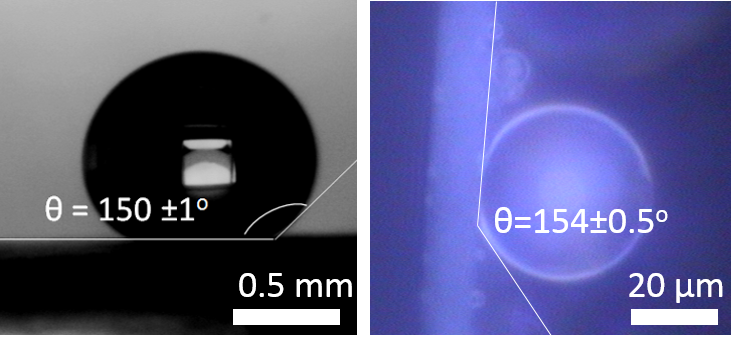  **Figure S2.** Contact angle of a ~40 µm droplet. |
| --- |

| 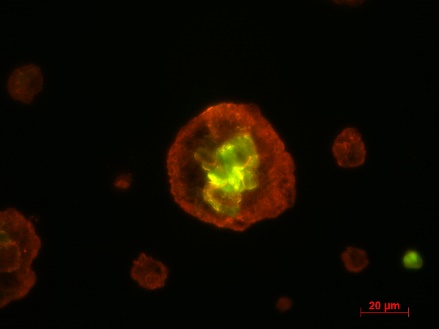  **Figure S3**. Representative morphologies of droplets exposed to a step change in RH from 100% to 29%. |
| --- |

| 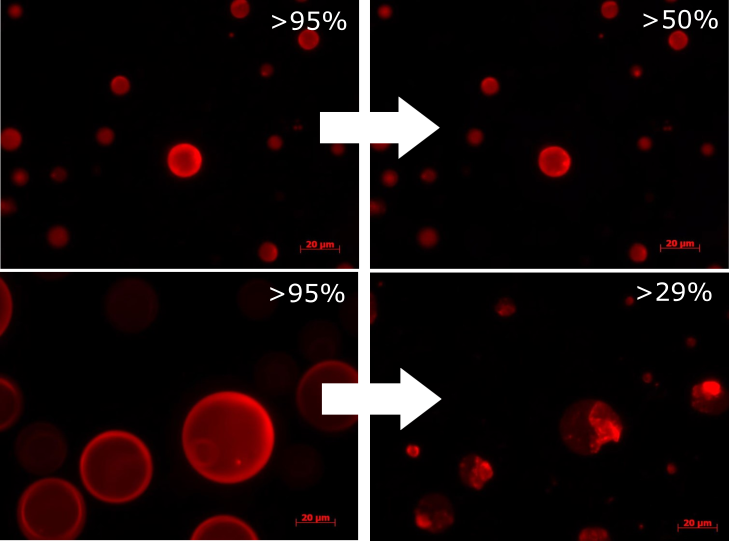 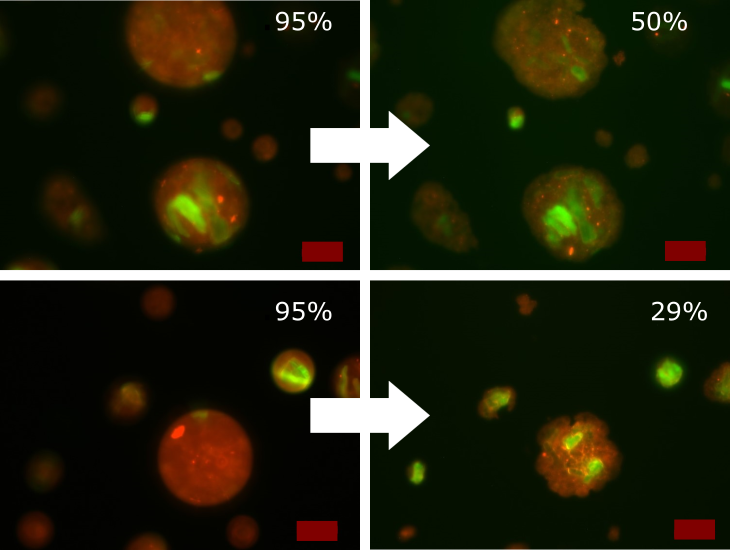  **Figure S4**. Phase separation in 3C (left) and 4C (right) droplets exposed at very high RH (≥95%) followed by a step change to 50% or 29% RH. Scale bar represents 20 µm. |
| --- |

| 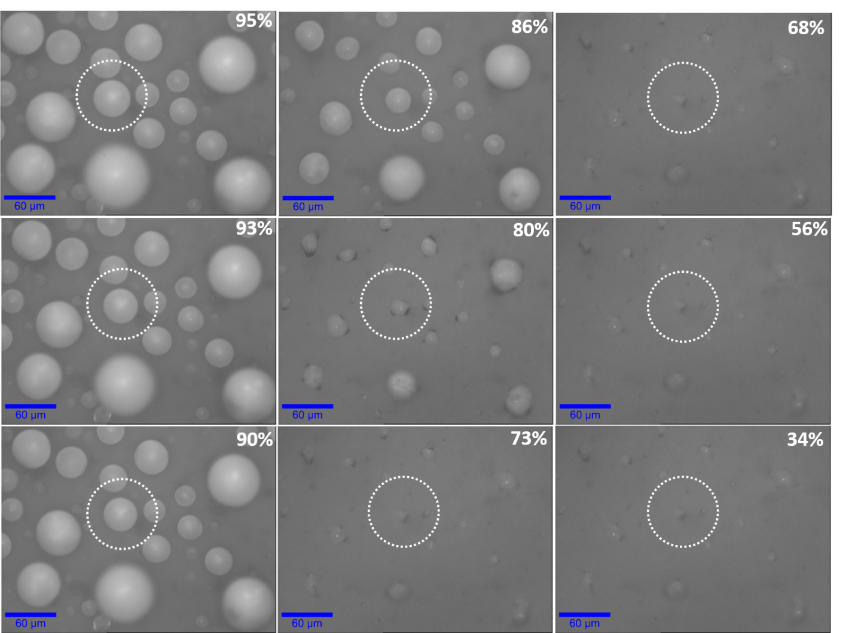  **Figure S5**. Sequence of confocal bright-field images for droplets exposed at different RH ramped down by 1% RH/min. The encircled droplet’s transparency changed at 80% RH. |
| --- |
